# Robotic Manipulation Planning for Automatic Peeling of Glass Substrate Based on Online Learning Model Predictive Path Integral

**DOI:** 10.3390/s22031292

**Published:** 2022-02-08

**Authors:** Liwei Hou, Hengsheng Wang, Haoran Zou, Yalin Zhou

**Affiliations:** 1 College of Mechanical and Electrical Engineering, Central South University, Changsha 410083, China; lwhou1992@gmail.com (L.H.); zouhr1995@163.com (H.Z.); 1012656597@163.com (Y.Z.); 2 State Key Laboratory for High Performance Complex Manufacturing, Central South University, Changsha 410083, China

**Keywords:** glass substrate peeling, manipulation planning, system model, deep learning, online learning, Model Predictive Path Integral

## Abstract

Autonomous planning robotic contact-rich manipulation has long been a challenging problem. Automatic peeling of glass substrates of LCD flat panel displays is a typical contact-rich manipulation task, which requires extremely high safe handling through the manipulation process. To this end of peeling glass substrates automatically, the system model is established from data and is used for the online planning of the robot motion in this paper. A simulation environment is designed to pretrain the process model with deep learning-based neural network structure to avoid expensive and time-consuming collection of real-time data. Then, an online learning algorithm is introduced to tune the pretrained model according to the real-time data from the peeling process experiments to cover the uncertainties of the real process. Finally, an Online Learning Model Predictive Path Integral (OL-MPPI) algorithm is proposed for the optimal trajectory planning of the robot. The performance of our algorithm was validated through glass substrate peeling tasks of experiments.

## 1. Introduction

**Figure 1:**
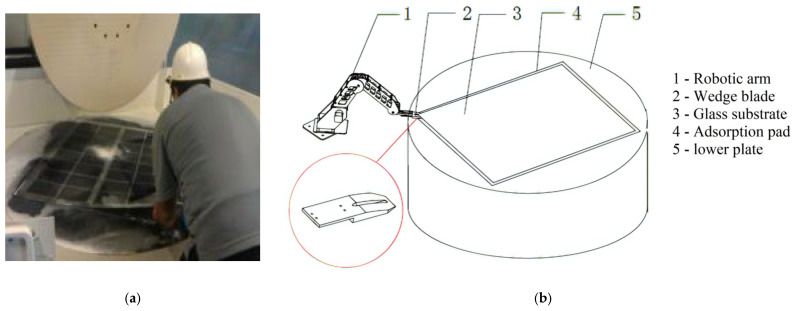
Unloading processes of glass substrate. (**a**) Manual unloading. (**b**) Automatic process setup.

The glass substrate is the base material of LCD flat panel displays, and its thinning process is necessary for the end product being lighter, thinner and smoother [1]. At present, the thinning process mainly adopts polishing machines with chemical mechanical processing. The workpiece of glass substrate, say 0.73 m in width, 0.92 m in length and 2 mm in thickness, needs to be fixed on the surface of a rotating rigid workbench plate, on which an adsorption pad is attached. The adsorption pad (a polyurethane foam pad about 1 mm thick) is generally used for the fixing task, and the glass substrate is absorbed into the pad with the vacuum suction force in between after pressing the glass substrate evenly on the pad. Separating the glass substrate from the pad is needed after the thinning process, and the process of unloading the glass substrate is usually done by human operators. They use their fingernails to “peel” the glass substrate off the pad from a corner of the substrate (Figure 1a). This manual process is labor-consuming, apt to glass damage, and therefore also expensive. Automating the process of unloading the glass substrate has long been expected, and we presented a scheme (Figure 1b) in [2]. It should be pointed out that the original intention of introducing Figure 1b here is to better show the task we need to accomplish. The actual experiment platform used can be seen in Figure 7, which is different from Figure 1b.

We invented a wedge blade (Figure 1b) as the end-effector of a robotic arm to imitate the “peeling” motion of human operators, but the action and reaction of human operators in the “peeling” process is still a challenge to imitate by robots, and this paper reports our work of the effort to make the robotic arm learn adaptively in the process to the end of replacing the human operator in the unloading process of glass substrate.

Our challenge is a kind of contact-rich manipulation in unstructured environments, which is one of the biggest challenges in robotics applications [3]. The external forces that the robotic arm faces during the peeling process is affected by the dynamics of the multi-interface contact, which shows a high degree of nonlinearity. The salient feature of the contact-rich manipulation task is that the robot or the tool at the end of the robot will remain in contact with the environment during the process. Such tasks are very common, such as assembly tasks (such as peg-in-hole task [4]) and material removal tasks (such as grinding [5], digging [6]) in the production environment, as well as glass cleaning [7], massage [8] and other tasks in life. There are mainly two kinds of ways for contact-rich manipulation tasks, namely, planning-based and learning-based. Learning-based methods such as reinforcement learning and imitation learning have been successfully applied in contact-rich tasks in recent years [9,10,11]. This type of method describes the manipulation tasks as a regression problem of machine learning, and establishes the mapping relationship from perceptual information to motion control signals. The advantage of learning-based methods is that they do not rely on prior knowledge, so as to improve the manipulation ability of robot in cross-task and cross-scene situations. However, the applications of learning-based methods have been almost accomplished in laboratory environments, and the robustness of the algorithms is still not enough for actual industrial application scenarios. This paper turns to planning-based method, which is another way of representing contact-rich manipulation tasks. The planning-based methods consider the manipulation planning as a motion planning problem with force constraints. The realization of planning-based methods includes two main stages: forward model establishment and planning.

There are many ways to derive the forward model, which can be divided into two types: mechanism model and data-driven model. The mechanism model relies on a large amount of prior information, which has a strong interpretability for the behavior of the robot [12]. The lack of generalization of the mechanism model makes it only suitable for specific task. For the glass substrate peeling process, it is almost impossible to establish an accurate mechanism model of the system, although we did make some effort on the process modeling. The pure data-driven method is to embed the physical system into the parameterized model without any prior information [13], but it needs a large number of training samples to make the model converge. As a result of this, pure data-driven modeling methods are not suitable for scenarios where sample collection costs are high, such as glass substrate peeling. Combining the mechanism model and the data-driven model is expected to bring out the advantages of both. The most classical method is parameter identification [14], but it still requires high modeling accuracy of mechanism models. Another classical method is to use a machine learning model to compensate the mechanism model [15]. When the mechanism model is inaccurate, this type of method requires the collection of sufficient experimental data to correct the mechanism model, which is also not suitable for the application scenarios of this paper. Both of the above two methods adopt the idea of mechanism modeling as the main part and data modeling as the supplement. Reference [16], on the other hand, takes the data modeling as the main part and mechanism modeling as the supplement. This modeling idea integrates the mechanism modeling as a priori information into the training process of the data-driven model, which significantly improves the training efficiency of the data-driven model. Reference [17] introduces the above modeling ideas into dynamic system modeling. The constraint of the methods in [16,17] is its only applicability to Newtonian rigid body systems, which is not the case of this paper that includes elastic objects such as glass substrate and adsorption pad.

Inspired by reference [17], this paper embeds the evolution of the physical system dynamics of peeling process of the glass substrate into the parameter model implicitly, and proposes a practical model structure and model training method. The specific description of the method will be introduced in Section 3.

When we complete the establishment of a forward model, it is needed to plan a control sequence that meets the task requirements according to the process. The planning of the control sequence for robot manipulations usually uses Linear Quadratic Regulator (LQR) [18,19] and Model Predictive Control (MPC) [20,21]. However, these methods require the differentiable overall loss function (or cost function) respect to the control variable, which makes they are not suitable for our cost function with contact force-related constraints, or to suit these methods we can only use force thresholds for safe operation which is a risky way to limit control inputs for safety.

Fortunately, path integral framework [22,23] allows more flexible design of cost function, and supports the planning directly with the nonlinear system forward model, rather than after linearization of the nonlinear model like LQR. The Model Predictive Path Integral (MPPI) planner proposed in reference [23] provides a suitable start for our manipulation planning of glass substrate peeling robot, but its offline nature [24] of system modeling cannot cover the uncertainty nature of our system. The contact dynamics of the glass substrate peeling process is greatly affected by the adsorption pad, the adhesive condition, and other working conditions, and the model from offline design may deviate greatly from the real process. This paper innovatively combines the meta-learning with MPPI to realize the online update of the forward model, thereby making the planner easily accommodate the uncertainty of the real process of the glass substrate peeling task while avoiding catastrophic forgetting of the model [25].

The main contributions of this paper are: (1) proposed a forward model modeling method that combines deep learning models and prior physical knowledge together; (2) proposed an algorithm based on Online Learning Model Predictive Path Integral (OL-MPPI), which is suitable for manipulation planning in tasks with large dynamical uncertainties.

The paper is organized as follows. Section 2 is a mathematical description of the research problem. Section 3 introduces the main algorithms. Section 4 is the experimental verification results of the algorithms. Finally, the conclusions are given in Section 5.

## 2. Problem Formulation

**Figure 2:**
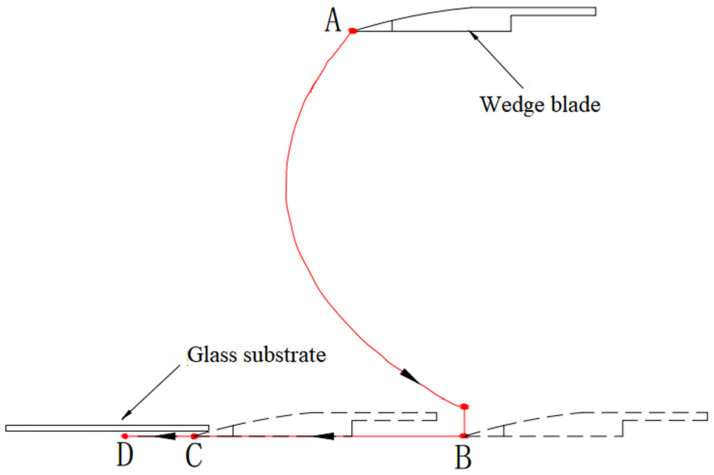
Schematic diagram of glass substrate peeling process.

The automatic process of the robotic arm in Figure 1b can be outlined in Figure 2: (1) From the initial point A to point B is an auxiliary motion, and at B, the wedge blade comes to the surface of the adsorption pad with a certain downward pressing force on the pad; (2) From B to C, the wedge blade keeps the downward pressing while moving horizontally until point C where it touches the edge of the glass substrate; (3) From C to D is the peeling process we are concerned about in this paper, after that other process and mechanical devices will be involved to separate the entire glass substrate from the adsorption pad (that is, to complete the workpiece unloading), which is beyond the scope of this paper.

This paper mainly considers the motion planning problem of the blade driven by the robotic arm from point C to point D. During this process, the wedge blade goes through the adhesive surface between the pad and the glass substrate to release the vacuum suction force. Our attention is mainly on the movement of the blade rather than the joints of the robotic arm, and the following movement descriptions will be in the task space.

Consider the following discrete system:

(1) $$x_{t+1}=f_{\theta }(x_{t},v_{t})$$

where $v_{t}$ is the wedge blade velocity at discrete time *t*, $x_{t}$ is the system state vector at time t which is defined in Equation (2), f parameterized by θ is the map of system state transition from time t to the next time point t+1. Equation (1) means that with the control input $v_{t}$ the system state transits from $x_{t}$ to $x_{t+1}$ according to the model defined by $f_{\theta }(x_{t},v_{t})$ with the set of model parameter θ.

Generally, the control variable $v_{t}\in R^{m}$. In our special case we only consider the horizontal movement of the blade from C to D, so m = 1. Here we model the peeling process as a discrete system, and actually this should be a top-level system model corresponding to kinematic control level, We will not involve bottom level of dynamical control in this paper, which should be our further experimental study about this project. We simply put $v_{t}\sim N(u_{t},\Sigma )$ to model the motion with acceleration and deceleration process of the real robot, where $u_{t}$ is the target velocity of the movement at control cycle t, and $v_{t}$ is the observation of $u_{t}$,with normal distribution, and Σ is the variance.

$x_{t}$ is defined as a triplet of position q, velocity v, and forces $F^{R}$:

(2) $$x_{t}=\left(q_{t-h:t},v_{t-h:t},F_{t-h:t}^{R}\right)\in R^{n}$$

where, $q_{t-h:t}$, $v_{t-h:t}$ and $F_{t-h:t}^{R}$ respectively represent the time series of position, velocity and contact force observations from time (*t* − *h*) to time *t*. *h* determines how much historical information is used to create the system model. The contact force of the wedge blade in the peeling process, $F_{t}^{R}$, is nonlinear and non-stationary in which only horizontal and vertical components $F_{t}^{RX}$ and $F_{t}^{RZ}$ are counted here.

A movement from point C to point D in Figure 2 is expressed as a trajectory τ, that is, $\tau =(x_{0},u_{0},x_{1},u_{1},\ldots ,x_{t},u_{t},\ldots x_{T})$, which is a time sequence of states and control inputs. where, $x_{0}$ and $x_{T}$ represent the initial state and the terminal state, and the sampling period of time between successive subscripts is Δt. In order to evaluate whether a trajectory $\tau_{i}$ meets the requirements of the glass substrate peeling task, we define the following cost function $J\left(\tau_{i}\right)$:

(3) $$J(\tau_{i})=\phi \left[x_{t_{T}},t_{T}\right]+\int_{t_{0}}^{t_{T}}L_{t}dt$$

where, $t_{0}$ is the initial time; $t_{T}$ is the terminal time; the cost of the trajectory includes two parts: cost obtained in the terminal state $\phi \left[\cdot \right]$, and the cost integrated through all the other steps; $L_{t}$ is called the immediate cost at time *t*, which is defined in Equation (4).

(4) $$L_{t}=-w_{dis}\frac{q_{t}-q_{t-1}}{q_{ter}-q_{0}}+w_{fX}\left|\frac{F^{RX}}{F_{\lim}^{RX}}\right|+w_{fZ}\left|\frac{F^{RZ}}{F_{\lim}^{RZ}}\right|$$

where, $q_{ter}$ and $q_{0}$ represent the end position and start position respectively; $F_{\lim}^{RX}$ and $F_{\lim}^{RZ}$ represent the maximum limit of horizontal and vertical force. It can be seen that the immediate cost function is composed of three items. The first one intends to encourage the robot to complete the task faster. The second and third items are used to punish the actions that cause excessive contact force and to reduce the wear or damage of the glass and the adsorption pad. Different task requirements can be achieved by adjusting coefficients $w_{dis}$, $w_{fX}$ and $w_{fZ}$.

When the robot does not reach the end position $q_{ter}$ within an upper bound time $t_{\max}$, the robot will get an additional terminal cost ϕ:

(5) $$\left\{\begin{array}{l} \phi =0,\;if\;(t<t_{\max}and\;q_{t}=q_{ter})\\ \phi =1,\;if\;(t>t_{\max}\;and\;q_{t}=q_{ter})\\ \phi =2,\;if\;(t>t_{\max}\;and\;q_{t}<q_{ter}) \end{array}\right.$$

According to the above definition, the manipulation planning problem of the glass substrate peeling robot can be regarded as a stochastic optimal control problem. The ultimate goal is to find the optimal control input sequence $\left[u_{0}^{*},u_{1}^{*},\ldots u_{T}^{*}\right]$ to minimize the value function:

(6) $$V(x_{0})=\underset{u_{0}:u_{T}}{\min}E_{\tau_{i}}\left[J\left(\tau_{i}\right)\right]$$

where, $E_{\tau_{i}}\left[\cdot \right]$ is the expectation of the cost function of all trajectories whose initial state is $x_{0}$.

## 3. Method

**Figure 3:**
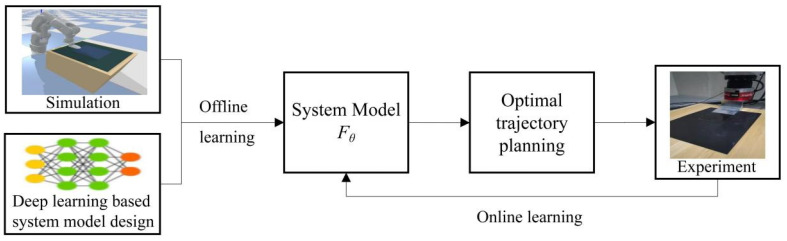
Manipulation planning framework for automated peeling of glass substrate.

Figure 3 shows our algorithm framework to solve the robot manipulation planning problem of automated glass substrate peeling formulated in Equation (6).

The two boxes of left-most in Figure 3 gives pretrained model, which is a deep learning-based neural network. In Section 3.1, we will introduce the design of deep learning model suitable for the glass substrate peeling task; simulation environment will be introduced (in Section 3.2) to collect samples for learning to avoid expensive and time-consuming collection of real data; the algorithm of offline learning of the deep neural network will be introduced in Section 3.3.

The loop in Figure 3 includes: (1) online learning (in Section 3.4) to tune the pretrained model according to the real data from the peeling process experiments to cover the uncertainties of the real process. (2) optimal trajectory planning based on the MPPI algorithm with the feature of online learning, which will be discussed in Section 3.5.

### 3.1. Model Design

We use deep neural network to model the discrete mapping function $f_{\theta }(*)$ in Equation (1), and sets the prediction range as one sampling period, that is, single-step prediction. Considering the observation range h>1 (in Equation (2)), the model of $f_{\theta }(*)$ should capture the nature of time sequence. The Recurrent Neural Network (RNN) and its variant Long Short-Term Memory (LSTM) [26] should be a reasonable choice for the model structure. The difficulties of the model training for our case comes from, (1) the noises of the input forces in Equation (2) which are from the real force sensor readings mounted on the end of the robotic arm, and (2) the uncertainties of the dynamic parameters of the peeling process.

The structure of the model proposed here in this paper can be depicted as in Figure 5, and we take several tricks to tackle the difficulties mentioned above and to improve the robustness and representation ability of the model. These tricks include the local feature extraction, the attention mechanism, and the time series feature extraction, and we connect these modules in series to obtain the final system model.

#### 3.1.1. Local Feature Extraction Based on Multi-Scale Convolution Kernel

The motion of wedge blade during the peeling process of the glass substrate takes the fashion of “stick-slip” motion. The proceeding forward of the wedge blade between the adsorption pad and the glass substrate needs the robot to drive the blade harder enough to overcome the resistance ahead. This resistance fluctuates very sharply, causing continuous stick-slip phenomenon in the movement of the blade. “Sticky” behavior is often concentrated on a small-time scale, while “slip” behavior is distributed in a large time scale. This feature can be extracted from time series data according to different time scales. Based on this, we designed a multi-scale 1D convolutional neural network (1D-CNN) to extract local features from time series data.

The convolutional layer uses the convolution kernel to traverse the input sequence at a certain step length. At each traversed position, use the following matrix convolution formula,

(7) $$y^{\left(i,j\right)}=w_{c}*x^{\left(i,j\right)}=\sum_{m}\sum_{n}x^{\left(i+m,j+n\right)}w_{c}{}^{\left(m,n\right)}$$

where, *w_c_* is the convolution kernel, and $x^{\left(i,j\right)}$ is the input series data whose starting point is (*i*,*j*).

**Figure 4:**
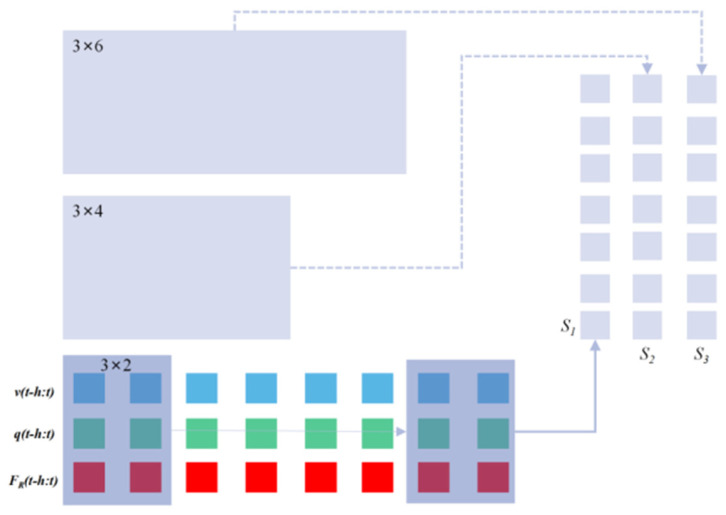
Local feature extraction based on multiscale convolution kernel.

For our application, we use three convolution kernels of different sizes, say 3 × 2, 3 × 4 and 3 × 6 respectively. The convolution kernels traverse the input sequence with stride = 1, and the paddings of the three convolution kernels are 0, 1, and 2, respectively to make the output sequence lengths consistent. The schematic diagram of extracting the local features of the input sequence with the multi-scale convolution kernel is shown in Figure 4.

#### 3.1.2. Attention Mechanism

Attention mechanism is used on the output sequences $S_{1}$, $S_{2}$, and $S_{3}$ in Figure 4 after the local feature extraction module. The attention mechanism is widely used in the fields of image and natural language processing. It can automatically assign different weights to the input vectors, so that the more valuable information for the prediction task can be fully used. The motivation for designing the attention mechanism is that when the blade is in different motion states, the importance of motion information and force information for interpreting contact dynamics should be automatically weighted. Intuitively, small changes in position or velocity should be weighted more than force information to infer the occurrence of the state switch from “stick” to “slide”, while from “slide” to “stick” the force information should count more. Considering the different importance of these three time series for force prediction, we design the following attention mechanism using *MLP* (a fully connected neural network) and SoftMax activation function:

(8) $$o_{t}=MLP([S_{1},S_{2},S_{3}])$$

(9) $$\left[w_{1},w_{2},w_{3}]=soft\max(o_{t}\right)$$

The output of the attention mechanism module can be calculated as follows:

(10) $$\left[S_{1}^{\prime },S_{2}^{\prime },S_{3}^{\prime }]=[w_{1}S_{1},w_{2}S_{2},w_{3}S_{3}\right]$$

#### 3.1.3. Time Series Feature Extraction Based on LSTM

The temporal dependence of physical variable is crucial for modeling contact dynamics, and the changing trends of different physical variables over time imply the next motion of the blade. We use LSTM neurons for this purpose.

The LSTM neuron contains a memory cell state and three gates: forget gate, input gate and output gate. In the time step of t, the formulas are as follows:

(11) $$f_{t}=\sigma (W_{f}[h_{t-1},x_{t}]+b_{f})$$

(12) $$i_{t}=\sigma (W_{i}[h_{t-1},x_{t}]+b_{i})$$

(13) $$g_{t}=\tanh(W_{g}[h_{t-1},x_{t}]+b_{g})$$

(14) $$C_{t}=f_{t}C_{t-1}+i_{t}g_{t}$$

(15) $$o_{t}=soft\max(W_{o}[h_{t-1},x_{t}]+b_{o})$$

(16) $$h_{t}=o_{t}\tanh C_{t}$$

where, f, *i*, *g*, *C*, and *o* are the forget gate, input gate, candidate update cell state, updated cell state, and output gate. tanh is the hyperbolic tangent activation function.

**Figure 5:**
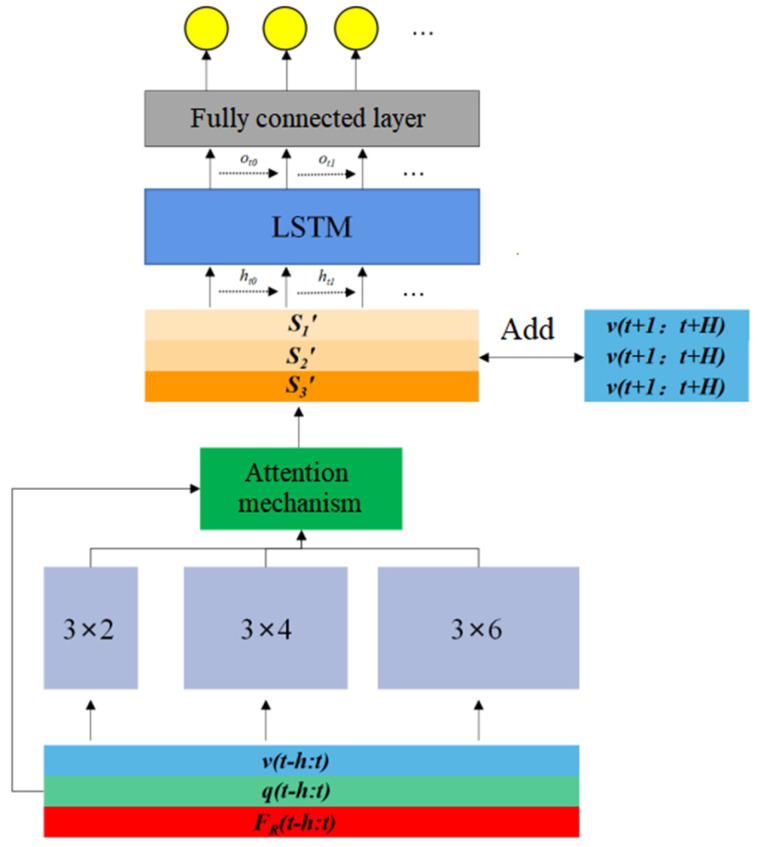
Structure of forward system model.

After concatenating the obtained feature vector with the velocity at the future time, then connect with the four-layer fully connected layer, so that we can get a complete system model based on deep learning. Here, the function of the fully connected layer is to realize the regression task. The number of neurons in each fully connected layer is 128, 64, 32, and 16, respectively, and the activation function uses the *ReLu* function. The overall structure of the model is shown in Figure 5.

### 3.2. Simulation Environment

Simulation can facilitate the process of our model learning. Based on the dynamics we studied previously [27], a simulation environment is established using Pybullet [28] as the physics engine to simulate the peeling process of the glass substrate.

There are uncertainties in the dynamic parameters of the glass substrate, the adsorption pad and the readings of force sensor, which have great influence on the accuracy of the simulation results. In order to make the simulation data more realistically reflect the real contact behavior, this paper adopts the following two measures:

#### 3.2.1. Dynamic Parameters Randomization

In the actual peeling process of the glass substrate, even if the same batch of adsorption pads are used, the contact behaviors between the blade and the adsorption pad may still be different due to the slight variations in the dynamic parameters. To improve the robustness of the offline training system model, this paper uses the method of dynamic parameters randomization to collect training samples containing different contact behaviors in the simulation environment. In this way, real contact dynamics information can be included in a part of the samples. *P_k_* = {*p*_1_, *p*_2_, …, *p_N_*}*_k_∈R^N^* represents a set of dynamic parameters, where *N* is the number of variable dynamic parameters. Different parameters combinations are sampled in the dynamic parameter space to construct different simulation environments, and each dynamic parameter *p*_i_(i = 1, 2,…, N) is uniformly sampled in the corresponding interval [*p*_i_^low^, *p*_i_^high^]. The variable dynamic parameters include the sliding friction coefficient between the glass substrate and the adsorption pad, as well as their contact stiffness and contact damping.

#### 3.2.2. Force Compensation

The contact forces in the peeling process are far from rigid. For example, the glass substrate is an elastic body, which will bend during the peeling process, and there is an adsorption force between the glass substrate and the adsorption pad. To reduce the gap between the simulation environment and the real environment, the influence of the above factors should be considered.

The force of the blade on the glass substrate, on the one hand, generates a shear force *f_bend_* that causes the glass substrate to bend, and on the other hand, it also provides the load *f_peel_* required for the desorption of the glass substrate.

The *f_bend_* can be calculated according to the deflection curve formula of the cantilever beam:

(17) $$f_{bend}=\frac{2EI\varphi }{L_{p}}$$

where *ϕ* is the desorption angle, *L*_p_ is the length of the glass substrate that has been peeled. the above two quantities can be observed in the simulation environment, and then calculated based on geometric relationship. *E* is the elastic modulus, and *I* is the bending moment of inertia.

Since the cross section of the glass substrate is rectangular, the thickness is *h*, and the width is *b*. Therefore, we have:

(18) $$I=\frac{bh^{3}}{12}$$

Substituting Formula (18) into Formula (17), we have:

(19) $$f_{bend}=\frac{Ebh^{3}\varphi }{6L_{p}}$$

Our previous studies have shown that the critical peel force ${\bar{f}}_{peel}$ for peeling the glass substrate from the adsorption pad is a function of velocity and peeling angle [28], and its physical unit is N. Combined with the measured data, the binary quadratic polynomial is used to approximate ${\bar{f}}_{peel}$, and the following expression is obtained:

(20) $$\begin{array}{l} {\bar{f}}_{peel}=0.1623+0.01834v\\ -0.001579\phi -0.0003526v^{2}\\ -6.707\times 10^{-6}v\phi +9.326\times 10^{-6}\phi^{2} \end{array}$$

We assume that each joint of the robotic arm that performs the task can provide a large enough torque to peel the glass substrate, so the robot can provide a driving force that meets the critical peeling force ${\bar{f}}_{peel}$ at any running speed, so $f_{peel}\approx {\bar{f}}_{peel}$.

Assuming that the inclination angle of the blade is α, the $F^{R}$ collected in the simulation environment can be compensated according to following equations:

(21) $${\tilde{F}}^{RX}=f_{bend}\cdot \sin\alpha$$

(22) $${\tilde{F}}^{RZ}=f_{bend}\cdot \cos\alpha +f_{peel}$$

where ${\tilde{F}}^{RZ}$ and ${\tilde{F}}^{RX}$ are the compensations for vertical and horizontal components of $F^{R}$, respectively.

### 3.3. Offline Learning of the Model

#### 3.3.1. Dataset Construction

The data obtained through simulation is stored in the form of trajectories. We adopt an experience replay mechanism [29] to obtain datasets that can be used for model training from these trajectory data. The motivation for adopting the experience replay mechanism is to allow the stored samples to be resampled multiple times to calculate the gradient. Compared with discarding after use, this mechanism of reusing past training data helps to improve sample efficiency.

Our experience replay mechanism is an adaptation of replay mechanism that is a one-step state transition $\left\{x_{t},v_{t},x_{t+1}\right\}$ and helps improve the exploration of reinforcement learning. To accommodate the time series signals in our application, our experience replay mechanism is based on trajectory slices. The experience pool stores a complete trajectory each time, and a fixed-length trajectory slice is intercepted from a random position of a random trajectory during sampling. This not only allows the model to learn the time series characteristics of the sample but also indirectly expands the sample size by randomizing the sampling trajectory and slice position.

**Figure 6:**
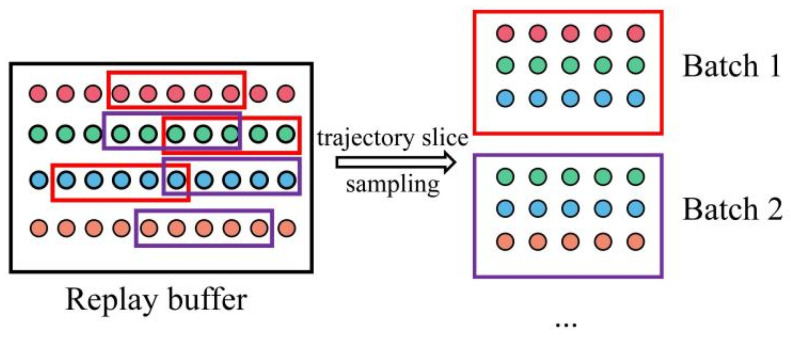
Sampling datasets based on a trajectory slice.

The training data sampling method used in the experience replay mechanism is shown in Figure 6, in which the trajectory slice is taken as the unit to extract data from the replay buffer to construct a batch of data, and then, the Mini-batch training method is used to train the system model.

#### 3.3.2. Loss Function

The establishment of the system model is through regression process, and the loss function is mainly the mean square error (*MSE*):

(23) $$L_{MSE}=\sum_{i=1}^{n}{\left[F\left(x,v\right)-{\hat{F}}_{\theta }\left(x,v\right)\right]}^{2}$$

where ${\hat{F}}_{\theta }\left(x,v\right)$ is the prediction of the model, and $F\left(x,v\right)$ is the ground truth.

An additional part of loss function is designed as follows:

(24) $$L_{PENE}=\sum_{i}^{n}\min{\left(0,\Psi_{\lim}-\psi_{p,n,i}\right)}^{2}$$

where $\psi_{p,n,i}$ is the distance that the blade is pressed into the adsorption pad, and $\psi_{\lim}$ is the limit distance that the blade is allowed to be pressed into. $L_{PENE}$ is added to avoid some unreasonable calculation results, which imply the wedge blade penetrating into the adsorption pad too deep.

The final regression of the system modeling is expressed as the optimization problem:

(25) $$\hat{\theta }=\underset{\theta }{agr\min}(L_{MSE}+\kappa L_{PENE})$$

where κ is the adjustment weight coefficient.

### 3.4. Online Learning of the Model

Since the training data collected in the simulation environment is inevitably different from the real data, the model through offline learning cannot be directly applied to online planning. Although we can fine-tune the model by collecting some samples online, it is impossible to directly construct a complete data set covering all the situations. The manipulation planning needs the model to be adjustable online.

The ordinary deep neural network has obvious shortcomings in solving online learning problems [30], for example, the inevitably catastrophic forgetting phenomenon [31]. When using new data for training, the updated neural network parameters would make the network tend to ignore the impact of past tasks, leading to the learning system catastrophically forgetting past knowledge. 

A straightforward idea is to store all past training samples [32]. Every time new samples are collected, the new samples and all past samples are used to update the model parameters. This will undoubtedly increase the data storage budget and increase the computation burden. More importantly, the new samples have a smaller proportion in the dataset, and the latest features of the system model are difficult to incorporate into the model parameter updates.

This paper introduces the idea of model agnostic meta learning (MAML) [33] for model training, trying to train a model with strong generalization ability, which can generally adapt to new peeling tasks. The algorithm is described below.

(1) A new trajectory $\tau_{new}$ is collected from an experiment, from which a dataset $D_{new}$ is sampled with the method shown in Figure 6. The parameters of the model are updated from θ to $\theta_{new}^{\prime }$ on the training set $D_{new}$, as follows with the gradient update step size α:
  (26) $$\theta_{new}^{\prime }=\theta -\alpha \nabla_{\theta }L_{D_{new}}(F_{\theta })$$
(2) From the experience pool (replay buffer) *M* trajectories, $\tau_{i}(i\in [1,M])$ are sampled corresponding to the *M* training set $D_{i}(i\in [1,M])$, and *M* sets of parameters $\theta_{i}^{\prime }(i\in [1,M])$ are obtained also through Equation (26).
(3) Another *M* + 1 dataset is sampled independently with $D_{New}^{\prime }$ from $\tau_{new}$ and $D_{i}^{\prime }(i\in [1,M])$ from $\tau_{i}(i\in [1,M])$. Now, the parameters are updated in a new way that sums up all the lost function together on this *M* + 1 dataset as follows. The purpose of this stage is to learn the commonality of all the trajectories.
  (27) $$\theta \leftarrow \theta -\beta \nabla_{\theta }\left(L_{D_{new}^{\prime }}\left(f_{\theta_{new}^{\prime }}\right)+\sum_{i=1}^{M}L_{D_{i}^{\prime }}\left(f_{\theta_{i}^{\prime }}\right)\right)$$
  where β is the gradient update step size. In the field of meta learning, $D_{new}$ and $D_{i}$ are also called support sets, and $D_{New}^{\prime }$ and $D_{i}^{\prime }$ are called query sets.

The pseudocode of the model online learning algorithm (Algorithm 1) based on meta learning is as follows:
**Algorithm 1**. Online learning of the model based on meta learningInputReplay Buffer *D*, Gradient update step size *α* and *β*1**While** not converge:2  Build data set $D_{new}$ from new trajectory $\tau_{new}$ collected in real time3  Obtain $\theta_{new}^{\prime }$ based on formula (26)4  Sample *M* trajectories $\{\tau_{1},\tau_{2},\ldots ,\tau_{M}$} from D5  **For**
*i* = 1 To *M*:6    Build data set $D_{i}$ from trajectory $\tau_{i}$
7    Obtain $\theta_{i}^{\prime }$ based on the formula similar to formula (26)8  Build data set $D_{i}^{\prime }$ from trajectory $\tau_{i}$
9  Build data set $D_{new}^{\prime }$ from trajectory $\tau_{new}$
10  Update *θ* according to formula (27)Outputθ

In step 4 of Algorithm 1, different sampling methods can be used. We use a task-oriented experience replay mechanism. First, use the trajectory scoring mechanism proposed in [34] to score and sort the trajectories. Then, we extract the M trajectories with the highest scores. This sampling strategy can introduce the task goal as a priori information into the model training.

### 3.5. Manipulation Planning Based on Online Learning Model Predictive Path Integral

After the establishment of the system model $x_{t+1}=F_{\theta }(x_{t},v_{t})$, the next step is to plan the robot motion according to the system model $F_{\theta }$ to complete the glass substrate peeling. Suppose the optimal distribution of the velocity for completing the task is represented by $Q^{*}(V)$. The optimal control input sequence satisfies:

(28) $$U_{t}^{*}=E_{Q^{*}}\left[v_{t}\right]=\int q^{*}(V_{t})v_{t}dV$$

$Q^{*}(V)$ is unknown, and we can use importance sampling to estimate the optimal control sequence:

(29) $$\int q^{*}(V_{t})v_{t}dV=\int \frac{q^{*}(V_{t})}{q(V_{t}\left|U,\Sigma \right.)}q(V_{t}\left|U,\Sigma \right.)v_{t}dV$$

We define ω(V) as the importance sampling weight:

(30) $$\omega (V)=\frac{q^{*}(V_{t})}{q(V_{t}\left|U,\Sigma \right.)}$$

Then Equation (28) can be written as:

(31) $$U_{t}^{*}=E_{Q_{U,\Sigma }}\left[\omega \left(V\right)\cdot v_{t}\right]$$

ω(V) can be split into two parts through the following transformation:

(32) $$\omega (V)=\left(\frac{q^{*}\left(V\right)}{p\left(V\right)}\right)\left(\frac{p\left(V\right)}{q\left(V\left|U,\Sigma \right.\right)}\right)$$

According to the information theory and optimal control principle, $J(\tau_{i})$ and the optimal velocity distribution $q^{*}(V)$, satisfy the following formula [35]:

(33) $$q^{*}(V)=\frac{1}{\eta }\exp\left(-\frac{1}{\lambda }J(\tau )\right)p\left(V\right)$$

(34) $$\eta =\int \exp\left(-\frac{1}{\lambda }J(\tau )\right)p\left(V\right)dV$$

Substituting Formulas (33) and (34) into Formula (32):

(35) $$\omega (V)=\frac{1}{\eta }\exp\left(-\frac{1}{\lambda }J(\tau )\right)\left(\frac{p\left(V\right)}{q\left(V\left|U,\Sigma \right.\right)}\right)$$

According to the probability density formula of Gaussian distribution, we have:

(36) $$\begin{array}{cc} \frac{p\left(V\right)}{p\left(V\left|U,\Sigma \right.\right)}= & \frac{\exp\left(-\frac{1}{2}\sum_{t=0}^{T-1}v_{t}^{T}\Sigma^{-1}v_{t}\right)}{\exp\left(-\frac{1}{2}\sum_{t=0}^{T-1}{\left(v_{t}-u_{t}\right)}^{T}\Sigma^{-1}\left(v_{t}-u_{t}\right)\right)}\\ = & \exp\left(-\frac{1}{2}\sum_{t=0}^{T-1}v_{t}^{T}\Sigma^{-1}v_{t}-v_{t}^{T}\Sigma^{-1}v_{t}+2v_{t}^{T}\Sigma^{-1}u_{t}-u_{t}^{T}\Sigma^{-1}u_{t}^{T}\right)\\ = & \exp\left(-\frac{1}{2}\sum_{t=0}^{T-1}2v_{t}^{T}\Sigma^{-1}u_{t}-u_{t}^{T}\Sigma^{-1}u_{t}^{T}\right) \end{array}$$

Since $v_{t}\sim N(u_{t},\Sigma )$, we can make $v_{t}=u_{t}+\varepsilon_{t}$. $\varepsilon_{t}$ is sampled from the normal distribution N(0,Σ), and the sampling algorithm used in this paper is Box-Muller transform [36]. Therefore, the above formula can be written as:

(37) $$\begin{array}{cc} \frac{p\left(V\right)}{p\left(V\left|U,\Sigma \right.\right)}= & \exp\left(-\frac{1}{2}\sum_{t=0}^{T-1}2u_{t}^{T}\Sigma^{-1}u_{t}+2\varepsilon_{t}^{T}\Sigma^{-1}u_{t}-u_{t}^{T}\Sigma^{-1}u_{t}^{T}\right)\\ = & \exp\left(-\frac{1}{2}\sum_{t=0}^{T-1}u_{t}^{T}\Sigma^{-1}u_{t}+2\varepsilon_{t}^{T}\Sigma^{-1}u_{t}\right) \end{array}$$

Therefore, ω(V) can be written as:

(38) $$\omega (V)=\frac{1}{\eta }\exp\left(-\frac{1}{\lambda }\left(J(\tau )+\frac{\lambda }{2}\sum_{t=0}^{T-1}u_{t}^{T}\Sigma^{-1}u_{t}+2\varepsilon_{t}^{T}\Sigma^{-1}u_{t}\right)\right)$$

In this way, the optimal control input sequence can be calculated according to Formula (31). Since the glass substrate peeling task is safety critical, a model predictive control framework is introduced; that is, only the first element of the optimal control input sequence is executed each time. The robot manipulation planning algorithm (Algorithm 2) for the glass substrate peeling process is summarized as follows:
**Algorithm 2.** Online Learning Model Predictive Path Integral (OL-MPPI)InputSystem model *F*_θ_, Number of sampled trajectories *N,* rollout steps *H*, *λ*InitialControl input sequence *U*, Variance *Σ*, Timing variable *t*, *D*_Σ_1While tasks not completed: 2  While *not (t > t_max_ or q_t_ > =q_ter_)*:3    x ← *GetStateFromSenors*()4    For k ← 0 To N-1:5      *J*_k_ ← 06      Sample $\varepsilon^{k}=\{\varepsilon_{0}^{k},\varepsilon_{1}^{k},\ldots ,\varepsilon_{H-1}^{k}\}$$,\;\varepsilon^{k}\in$ N(0,Σ)7      For *t*← 0 To *H*-1:8        *v*_t_ = *u*_t_ + *ε*_t_9        *x* ← *x* + *F*_θ_(*x*,*v*_t_)∆*t*10        Obtain *L*_t_ according to Formula (4), *J*_k_←*J*_k_ + *L*_t_11      If *q_t_ > =q_ter_*:12        Obtain ϕ according to Formula (5), *J*_k_←*J*_k_ + ϕ13        Obtain $\omega_{k}$ according to Formula (38)14      Update *U* based on formula (31)15    Execute u* = *U*_0_ and Measure v*, *D*_Σ_ ←{v*-u*}16    
t←t+Δt
17    Update *Σ* with *D*_Σ_18    For *t* ← 0 To *H*-1:19     *u*_t-1_ ← *u*_t_20  Update *θ* with Algorithm 1, *t*←0

## 4. Experiments

### 4.1. The Experiment Platform

**Figure 7:**
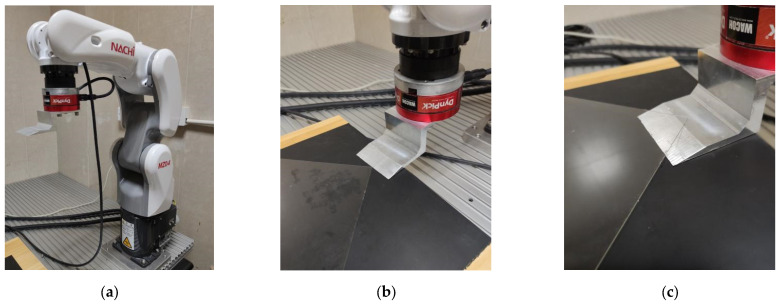
Experiment platform. (**a**) Layout of the system. (**b**) Starting point of peeling. (**c**) End of peeling.

**Figure 8:**
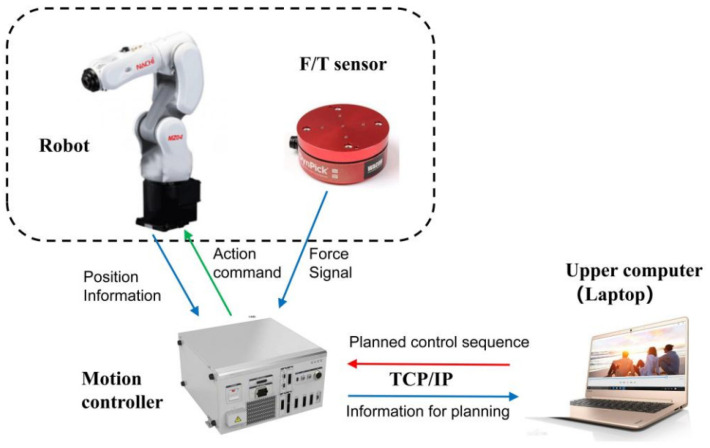
Experiment system layout.

(1) The experimental platform is shown in Figure 7. Figure 7a is the Nachi 6DOF robot MZ04 with about ±0.1 mm of position tracking accuracy. It should be noted that the position tracking accuracy may deteriorate in the contact task environment. A six-dimensional force/torque sensor WEF-6A is installed at the end of the robot. The rated load of the sensor in three directions is ±500 N, and its measurement accuracy is 0.01 N. Figure 7b,c respectively show that the blade is located at the starting point and the end point of the peeling task. The thickness of the glass substrate we used is 0.5 mm, and the total distance that the robotic arm needs to move forward is 25 mm, that is, $q_{\mathrm{ter}}=25\;\mathrm{mm}$.
(2) We conducted simulation and offline model training on a computer server equipped with a GTX 1080Ti GPU.
(3) A laptop computer was used for all the online algorithms calculation of experiments, which is connected to the robot controller with Ethernet TCP/IP protocol. The laptop CPU is Intel Core i7 8550U, and the GPU is NVIDIA Geforce MX150.
(4) In the experiments, the robot works in speed control mode. The laptop calculates the optimal control speed and sends it to the robot controller for execution. The layout of the system is shown in Figure 8.

### 4.2. Performance Measures

(1) System Model Evaluation

We use the root mean square error (*RMSE*) and correlation coefficient (*Corr*) as the evaluation index of the system model. The former can be used to judge the deviation of the predicted results, and the latter can be used to test whether the predicted trend of the model is consistent with the real trend. *RMSE* and *Corr* can be calculated according to the following formula:

(39) $$RMSE(y)=\sqrt{\frac{1}{n}\sum_{i}^{n}{\left(y_{i}-{\hat{y}}_{i}\right)}^{2}}$$

(40) $$Corr(y,\hat{y})=\frac{Cov(y,\hat{y})}{\sqrt{Var(y)Var(y^{\prime })}}$$

where *y* and $\hat{y}$ represent the real measured value and the model predicted value of the variable, respectively, and Var(y) and $Var(\hat{y})$ represent the variance of the real value and the predicted value of the variable, respectively.

(2) Planning Results Evaluation

Two merits, namely the speed and the contact forces, are used to measure the performance of the peeling task, from C to D (Figure 1). The higher the speed to complete the task and the smaller the contact forces during the peeling, the better the performance is. The total cost function calculated according to Formula (3) can comprehensively reflect the above two aspects. Therefore, the cost function $J(\tau_{i})$ is used as an index to evaluate the overall performance of the planning result.

In addition, the task completion time tcpl is also used to evaluate the task completion speed, and the sum of the contact forces $\sum F^{RX}$ and $\sum F^{RZ}$ of all sampling points is used to evaluate the wear of the blade and pad. In the experiments, the sampling period is 100 ms, and the control period is 500 ms.

To avoid robot jamming at a certain position, set the maximum task completion time $t_{\max}=60\;s$. To avoid irreversible damage to the workpiece caused by the motion of the manipulator, the safety prewarning value of the horizontal and vertical contact force is set to 20 N during the peeling process. In all experiments, when the contact force exceeds the prewarning value, or the time exceeds $t_{\max}$, the experiment will be immediately stopped.

### 4.3. Model Offline Learning Process and Results

A simulation environment was constructed in Pybullet, and a total of 1000 sets of dynamic parameters were set to carry out simulation experiments. According to each group of dynamic parameters, 50 trajectories were sampled, and finally, a total of 50,000 trajectories were collected. Among them, 40,000 trajectories were used as the training set, 5000 trajectories were used as the verification set and the remaining 5000 trajectories were used for testing. Obtain the triple $\left(x_{t},v_{t},x_{t+1}\right)$ from the trajectory as a training sample, where $x_{t}$ is also a triple; see Formula (2). Among them, let the speed $v_{t}$ be equal to the ratio of the robot movement distance to the sampling period from time *t* to time (*t* + 1) and other quantities can be directly observed. The deep learning model is constructed by Pytorch. The optimizer used to solve Formula (25) is Adam, and the learning rate is equal to 0.0001.

**Figure 9:**
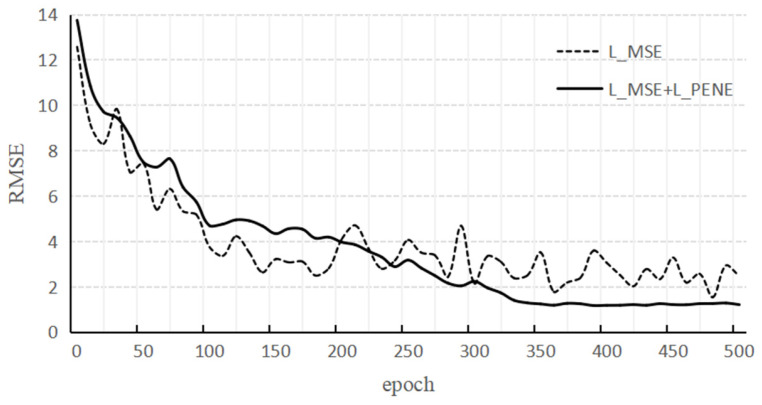
RMSE curve under different epochs.

Figure 9 is the RMSE curve under different epochs. The dotted line in the figure is the case where only *L_MSE_* is used, and the solid line is the case where both *L_MSE_* and *L_PENE_* are used. It can be found that the model performance jitter is obvious when only *L_MSE_* is used, while training is more stable when *L_MSE_* and *L_PENE_*. The reason for this phenomenon is that the former will fit some simulation data that is contrary to the actual physical situation, and the distribution between this type of data and the real data is very different, so the model training is unstable.

**Figure 10:**
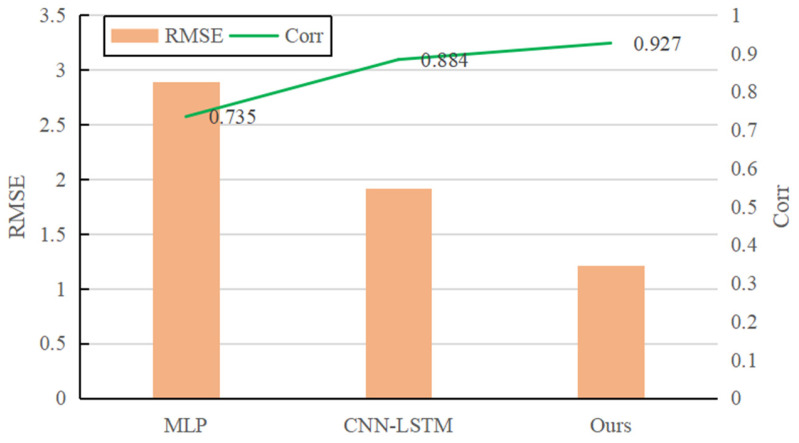
Performance comparison of different models.

After offline training of the model based on simulation data, the *RMSE* is 1.21 after the model converges. To verify the superiority of the system model designed in this paper, the model is compared with the MLP model [37] and CNN-LSTM model [38], which are commonly used in data-driven system modeling. In the experiment, the MLP model and the regressor of our model adopt the same structure. The CNN-LSTM model only uses a single-size convolution kernel, and other settings (including the attention mechanism module) are consistent with the model we propose. The comparison results of the three models are shown in Figure 10. It can be seen that the prediction performance of the model in this paper is better than that of the MLP model and the CNN-LSTM model. The multi-scale convolution kernel used in this model can extract different levels of local information. Compared with the single-size convolution kernel model, it has obvious advantages in the representation of contact dynamics.

### 4.4. Manipulation Planning Results

In each peeling experiment, the planner will simultaneously simulate *N* = 50 trajectories based on the observation after each step of the control command and calculate their weights. Then, a new control sequence is obtained based on the weights, and the first step of the control sequence is executed. To test the robustness of the method proposed, 20 consecutive peeling experiments were carried out, during which there was no glass substrate breakage. As a comparison, we compared two other baseline methods:

1. Baseline 1: The system model is not updated, as in the original MPPI.
2. Baseline 2: Compared with OL-MPPI, each update of the system model uses all previous historical experience.

**Figure 11:**
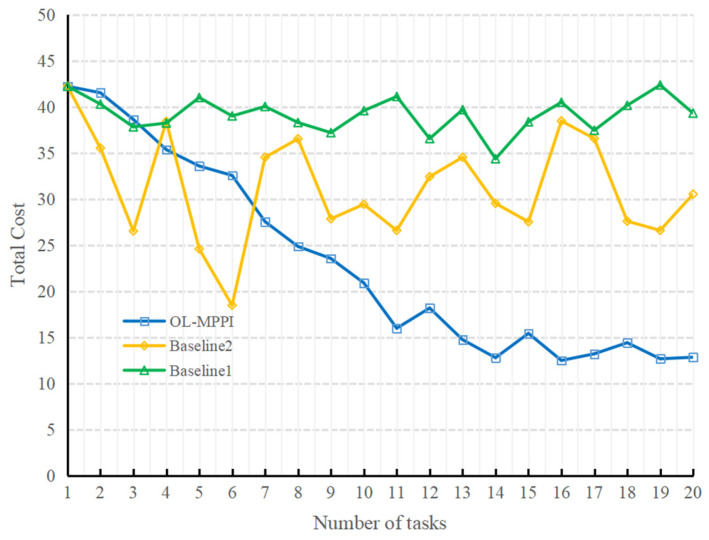
Total cost of different planning methods.

The total cost of the three methods for 20 consecutive peeling experiments is shown in Figure 11. According to the results of Baseline1, it can be seen that the total cost of the 20 peeling experiments is relatively stable, which shows that the experiments in this paper have good consistency. From the results of OL-MPPI, it can be seen that, as the number of times of performing tasks increases, the total cost decreases significantly, reduced from 42.22 in the 1st task to 15.98 in the 11th task. It shows that, as more trajectory data containing real system dynamics is accumulated, the system model can better characterize the real manipulation environment. In contrast, if the system model is trained with all previous experience each time, the total cost fluctuates greatly. Although the cost was reduced to 18.46 in the sixth experiment, it was difficult to maintain good performance due to catastrophic forgetting. In summary, the OL-MPPI algorithm can gradually improve the quality of manipulation planning.

Table 1 shows the task completion time and total contact force of the 20th peeling task. It can be seen that the algorithm proposed in this paper can complete the task in the shortest time and can significantly reduce the total wear on the glass substrate.

**Figure 12:**
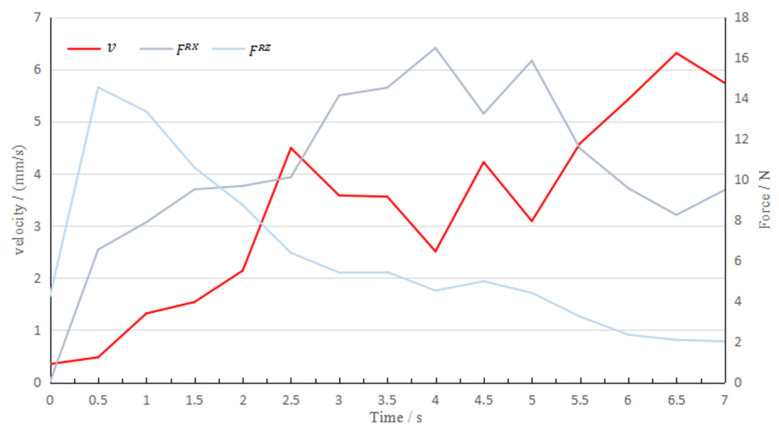
Velocity and the force in the two directions over time in the 20th task.

Figure 12 shows the curve of the robot velocity and the force in the two directions over time in the 20th task. 

When the peeling starts, the horizontal and vertical forces fluctuate greatly. At this time, the velocity is slow to avoid glass breakage due to excessive contact force. As the vacuum between the glass substrate and the adsorption pad is broken, the horizontal force becomes the main factor that determines the velocity of the robot. At 3.0 s, 4.0 s and 5.0 s, the increase in horizontal contact force is accompanied by a decrease in the planned velocity. It can be seen that the planned control input sequence allows the robot motion to balance efficiency and wear, and its motion behavior is very similar to that of experienced workers.

### 4.5. Hyperparameter Analysis

The OL-MPPI algorithm can meet the needs of automatic peeling of glass substrates. However, in the experiment, we found that the hyperparameters in the OL-MPPI algorithm have a certain impact on the performance, which we will discuss here. We mainly focus on the influence of the three hyperparameters of *N*, *H* and *λ*. Using the system model used in the 20th peeling experiment, different hyperparameters were selected to carry out comparative experiments. The results are shown in Table 2.

- *N* represents the number of simulations based on the system model in a single plan. Since the system model is highly nonlinear, it may not be possible to explore the state with low cost with a small number of simulations. When the computing resource is sufficient enough, sampling more trajectories can theoretically achieve better results. The experimental results show that the total cost decreases as *N* increases, which is consistent with our expectations. However, it should be noted that, when the value of *N* exceeds a certain threshold, the reduction in cost becomes smaller. Although the experiments were carried out with limited computing resources, choosing the right *N* can still achieve good performance.
- *H* determines the length of the forward simulation. A higher *H* means that the agent has a “broader vision” of the task. However, as *H* increases, it will inevitably spend more time and may even fail to complete the calculation within the control period. It can be seen from the experimental result that with the increase of *H*, the total cost shows a trend of first decreasing and then increasing. This is because if *H* is selected too small, then the agent will not know the future loss, and the decision is too short-sighted. Setting *H* too large may amplify the error of the system model and then formulate unreasonable planning results based on the simulation results with large deviations.
- *λ* determines the importance of the trajectory with the lowest cost. When *λ* is extremely small, the final trajectory will almost be the lowest cost trajectory, which easily makes the control sequence a local optimal solution. If *λ* is too large, the algorithm will be similar to random shooting. The experimental results show that there is a nonlinear relationship between the value of *λ* and the total cost. However, in general, when *λ* is within a certain range, the manipulation performance is not sensitive to *λ*.

## 5. Conclusions

Contact-rich manipulation is a challenging problem in robotic engineering applications. This paper takes the automated peeling of glass substrates as an example and proposes the OL-MPPI algorithm. 

The proposed algorithm can perform manipulation planning in real time by updating a forward system model online. We carried out experiments to verify that the control sequence planned can meet the demand for automated peeling of glass substrates. The OL-MPPI algorithm can also be applied to other application scenarios involving contact-rich manipulations, because only a small amount of prior information about the task needs to be used to accelerate the offline model learning process, and the rest of the algorithm is scenario-independent. The advantage of the OL-MPPI algorithm is not only that it can support flexible cost function design but also can adapt to changes in the environment. This paper also provides some tricks that combine analytical mechanics and data-driven methods to build complex system models, which can be applied to various nonlinear dynamical systems in engineering applications. The forward model in the OL-MPPI algorithm can also use the pure mechanism model. In the online learning part of the OL-MPPI model, the mechanism model can be corrected online by adding a compensation term.

In the future, we will further improve the OL-MPPI algorithm and make efforts from the following aspects: (1) improve the efficiency of trajectory sampling in the planning process, so that the performance of the algorithm will no longer depend on the GPU used and (2) theoretically prove the influence mechanism of hyperparameters on the performance of the algorithm and provide a basis for the hyperparameters setting.

## Figures and Tables

**Table 1:** Duration and wear of the peeling task.

Method	*T*_cpl_ (s)	${\sum F^{RX}}_{\left(N\right)}$	${\sum F^{RZ}}_{\left(N\right)}$
Baseline1	13.5 s	1388.48 N	836.37 N
Baseline2	10.4 s	1057.54 N	612.41 N
OL-MPPI	7.1 s	784.64 N	486.45 N

**Table 2:** The impact of hyperparameters on performance.

Hyperparameter	Value	Total Cost
*N*	10	18.614
	30	15.448
	50	12.834
	80	12.226
	100	12.098
*H*	1	24.691
	3	17.541
	5	12.834
	7	16.657
	10	20.228
*λ*	0.02	13.178
	0.05	12.421
	0.1	12.834
	0.2	12.086
	0.5	13.551

## Data Availability

Not applicable.
